# Germanium-Titanium-π Polymer Composites as Functional Textiles for Clinical Strategy to Evaluate Blood Circulation Improvement and Sexual Satisfaction

**DOI:** 10.3390/polym13234154

**Published:** 2021-11-27

**Authors:** Yu-Cing Juho, Shou-Hung Tang, Yi-Hsin Lin, Chen-Xi Lin, Tenson Liang, Juin-Hong Cherng, En Meng

**Affiliations:** 1 Division of Urology, Department of Surgery, Tri-Service General Hospital, National Defense Medical Center, Taipei 114, Taiwan; wavinglibra1012@gmail.com (Y.-C.J.); tansohorn@gmail.com (S.-H.T.); 2 Department of Obstetrics and Gynecology, Tri-Service General Hospital, National Defense Medical Center, Taipei 114, Taiwan; M860371@gmail.com; 3 School of Nursing, National Defense Medical Center, Taipei 114, Taiwan; lin050027@hotmail.com; 4 Quality of Pain and Sleep Association, Taipei 105, Taiwan; tenson88888@gmail.com; 5 Graduate Institute of Life Sciences, National Defense Medical Center, Taipei 114, Taiwan; 6 Department and Graduate Institute of Biology and Anatomy, National Defense Medical Center, Taipei 114, Taiwan

**Keywords:** bioactive polymers, polymer composites, arteriovenous fistula, blood flow, erectile dysfunction, hemodialysis

## Abstract

By continuously enhancing the blood flow, far-infrared (FIR) textile is anticipated to be a potential non-pharmacological therapy in patients with peripheral vascular disorders, for instance, patients with end-stage renal disease (ESRD) undergoing hemodialysis (HD) and experiencing vasculogenic erectile dysfunction (VED). Hence, we manufactured a novel polymer composite, namely, germanium-titanium-π (Ge-Ti-π) textile and aimed to evaluate its characteristics and quality. We also investigated the immediate and long-term effects of the textile on patients with ESRD undergoing HD and experiencing VED. The Ge-Ti-π textile was found to have 0.93 FIR emissivity, 3.05 g/d strength, and 18.98% elongation. The results also showed a 51.6% bacteria reduction and negative fungal growth. On application in patients receiving HD, the Ge-Ti-π textile significantly reduced the limb numbness/pain (*p <* 0.001) and pain score on the visual analog scale (*p <* 0.001). Moreover, the Doppler ultrasound assessment data indicated a significant enhancement of blood flow in the right hand after 1 week of Ge-Ti-π textile treatment (*p <* 0.041). In VED patients, the Ge-Ti-π underpants treatment significantly improved the quality of sexual function and increased the average penile blood flow velocity after 3 months of the treatment. Our study suggests that the Ge-Ti-π textile could be beneficial for patients with blood circulation disorders.

## 1. Introduction

Bioactivity is the ability of a particular substance to elicit effects, such as tissue uptake, metabolism, or physiological response, in biological surroundings [1,2]. Macromolecules that are bioactive and cause a definite biological response when used for medical therapy, insect control, weed control, or other reasons are called bioactive polymers [3,4]. Some polymer composites are used to improve the characteristics of certain materials. A polymer composite is a multi-phase material wherein the polymer acts as a matrix for reinforcing fillers; this material structure leads to synergistic mechanical traits that may be impossible to achieve if only one material is used [5]. Composites have received much traction because of their promising properties such low weight, corrosion resistance, and excellent fatigue strength [6]. Moreover, polymer composites are commonly employed in biomedical practices in several fields, such as regenerative medicine, restorative dentistry, and tissue engineering [7,8]. The combination of polymers as matrices and active materials as fillers (e.g., germanium, magnesium oxide, titanium dioxide, bamboo charcoal, or nephrite) can make polymer composites emit far-infrared (FIR) that may be useful for clinical therapy. In the electromagnetic spectrum, FIR has a wavelength of 15 μm to 1 mm, which falls within the infrared wavelength range (750 nm–1 mm) [9]. FIR is beneficial for medical treatment because in the IR wavelength, only FIR produces gentle radiant heat and has high penetrability (almost 4 cm) that can be sensed by local thermoreceptors of skin [10,11]. As FIR can produce thermal effects on the body, it can be utilized to elevate local tissue temperature that leads to blood vessel dilation; this mechanism could be used to improve blood circulation in active areas of the body [12]. Besides its thermal effect, FIR can also inhibit intimal hyperplasia, reduce oxidative stress, and suppress vascular endothelial inflammation [13,14,15].

Endothelial dysfunction refers to the impaired functioning of the blood vessel lining, resulting in vascular lesions, inflammation, thrombosis, and atherosclerosis [16,17]. It often causes micro- or macro-vascular disruption of the blood flow, a prevailing problem observed in patients with end-stage renal disease (ESRD) undergoing hemodialysis (HD) and experiencing vasculogenic erectile dysfunction (VED) [18]. Several common risk factors, such as smoking, diabetes mellitus, hyperlipidemia, and obesity, are prevalent and causative in patients with endothelial dysfunction; thus, it is likely that metabolic factors play a crucial role in the disease pathogenesis [19].

In patients with ESRD undergoing HD, the creation of an artificial arteriovenous fistula (AVF) is one of the most commonly performed surgeries, and a sufficient increment of blood flow and dilatation of vessels is required for the AVF to be functional for HD [20,21]. VED, however, is the persistent incapability to achieve and maintain an adequate penile erection, thereby resulting in unsatisfactory sexual performance that considerably affects the patients’ quality of life [22]. During an erection, blood fills the lacunar spaces in the corpora cavernous, initiating the distention and elevation of the penis [23]. Thus, the maintenance of adequate blood circulation is essential for a successful AVF and penile erection.

Various options for optimizing blood flow and circulation are available, including lifestyle modifications, oral and parenteral drugs, injectable vasodilator agents, surgery, and/or psychosexual therapy. Among these treatments, FIR can be considered as a promising therapeutic approach in maintaining vascular endothelial health and function [24]. Additionally, FIR therapy can enhance blood flow and maintain unassisted AVF patency in patients undergoing HD [25]. FIR is also shown to increase endothelial nitric oxide synthase (eNOS) activity and the nitric oxide (NO) concentration in cells [24,26,27]. Since NO is the main neurotransmitter regulating a penile erection and is released from the vascular endothelium [23,28], increased eNOS expression and NO production are predicted to mediate the recuperation of impaired vascular endothelial function in patients with VED.

Textiles are considered an effective device for localized treatment of the human body. In recent years, advances in the textile industry and in nanotechnology have resulted in the development of high-technology textiles that can emit FIR, making them convenient treatment devices that could benefit the body by locally promoting blood circulation and improving metabolism [29,30]. However, several factors, such as wavelength, endurance, timing, and treatment repetition, influence the therapeutic effects of FIR. Therefore, textiles with active FIR materials that can generate persistent ray stimulation during the treatment period are needed. Conjugated polymers are commonly used as functional textiles because these materials have a backbone of alternating single and multiple bonds forming a π-conjugation by overlapping π-orbitals producing a continuum of energy states; the π-conjugated backbones provide a semiconducting property [31,32]. Therefore, π-conjugated polymers can be used to create textiles that can emit preserved FIR. This technology can transform materials with electrical characteristics and polymers into functional textiles.

As an active material, germanium fibers can release negative ions that have a positive effect on the human body because of their semiconducting properties. When germanium is exposed to temperatures above 32 ℃ or altered atmospheric pressure, one of its unstable electrons may be excited from the outer orbit and ionize the surrounding air, resulting in negative air ions [33]. Hence, if germanium is used in certain garments, the negative air ions could be easily activated by the human body temperature (36.5 ℃) and the frequent alteration in atmospheric pressure caused by limb movement; thus, textiles doped with germanium could continuously generate negative air ions [34]. The germanium ions that penetrate the human skin and are transported in the blood can permeate the capillary inside epidermal tissues. These ions can promote ionic equilibrium in the blood, the activation of biologic energy, and the improvement of blood circulation that may relieve physical discomfort [35,36]. In addition, the negative ions released by germanium also emit FIR radiation that is beneficial for clinical therapy [36]. Moreover, Yang J. and Liu Y. revealed that the germanium fiber yarn is safe and compatible for use as a next-to-skin textile product [35]. Although Chen Z et al. found that combining PET and germanium into composite fibers showed remarkable FIR emission traits, they also reported that increasing the concentration of germanium could lead to a lower fiber breaking strength [34].

Ceramic particles such as titanium oxide (TiO_2_) can emit FIR persistently [37]. When incorporated into a textile, ceramic particles promote excellent anti-bacterial characteristics as a coating material [38]. Studies have confirmed the bacterial inhibition and self-cleaning features of TiO_2_ nanoparticles in polyester fabrics [39,40]. In a study on the addition of ceramic particles to polyurethane films, Faisal et al. showed that TiO_2_ has the highest FIR emissivity among other ceramic particles such as Al_2_O_3_. The study also exhibited that TiO_2_-embedded PU films have a comparable contact angle and mechanical properties to Al_2_O_3_- and SiO_2_-doped PU films [41]. In biomedical applications, titanium combined with polyimide is successfully used as an implant material owing to the biocompatibility of the components [42]. In addition, according to previous studies, the augmentation of TiO_2_ as a filler to a polyimide matrix could improve the insulation property of polyimide-based composites [43,44]. Increasing the insulation property could preserve FIR emission and promote thermal stability in a functional textile [30].

Considering the properties of germanium and TiO_2_ mixed with polymers and the ability of the combined material to emit FIR emission effectively, we created a novel polymer composite called the germanium-titanium-π (Ge-Ti-π) textile. In this study, we aimed to evaluate the characteristics and quality of the Ge-Ti-π textile, and further investigate the immediate and long-term effects of the textile on patients with ESRD undergoing HD and experiencing VED. To the best of our knowledge, the effectiveness of a continuous FIR-emitting textile in treating blood circulation in these patients has not been clearly elucidated.

## 2. Materials and Methods

### 2.1. Preparation of the Ge-Ti-π Textile

The experimental textiles, made from pure Ge-Ti metals combined with polymers, Tencel, and cotton fibers, were manufactured by the Taiwan Textile Research Institute, New Taipei, Taiwan and Green Energy Nano Technology Co., Ltd., Taipei, Taiwan. The manufacturer claimed that the Ge-Ti-π textile can generate FIR without sagging, losing its luster, appearing deformed, or stiffening after washing. The ingredients and the manufacturing process are briefly described as follows.

#### 2.1.1. Preparation of Far-Infrared Masterbatch

A fiber masterbatch was prepared using an FIR filler and a polymer matrix. The FIR filler contained the following elements: titanium (Ti): 5~40 wt%, germanium (Ge): 0.01~1.0 wt%, zinc (Zn): 1~12 wt%, aluminum (Al): 3~16 wt%, and magnesium (Mg): 1~15 wt%. Meanwhile, the first polymer matrix of the FIR masterbatch included polyester, polyurethane (PU), poly vinyl chloride (PVC), polypropylene (PP), polyamide (PA), and amino-containing polymers (Polyethylenimine (PEI)). Thereafter, using either polyethylene terephthalate (PET) or polybutylene terephthalate (PBT), the first FIR filler, the first polymer matrix, and the dispersant were mixed in a high-speed mixer. Then, the FIR masterbatch was manufactured by blending and extruding the mixed ingredients at 245~290 °C using an extruder.

#### 2.1.2. Preparation of Far-Infrared Fibers and Textiles

The FIR fibers were obtained by mixing the FIR masterbatch and the second polymer matrix including polyester, PU, PVC, polypropylene (PP), polyamide (PA), and polysiloxan in a weight ratio of 1:9. Furthermore, a melt spinning process was used to extrude the FIR masterbatch and the second polymer matrix into an FIR fiber. The extrusion was performed at 245~265 °C, and since the FIR masterbatch and the second polymer matrix reached the fluidity phase during the extrusion process, the first FIR filler could be evenly distributed to form core-shell fibers. In the melt spinning process, the molten fibers produced were quenched after the ingredients were melted and extruded. Following this, the solid fibers underwent stretching and winding until the spun FIR fibers were obtained [45]. The melt spinning process was utilized because of its economic and ecological benefits among other spinning processes [46]. Another benefit of this process is that it generates FIR fibers through high-speed production [47].

**Figure 1:**
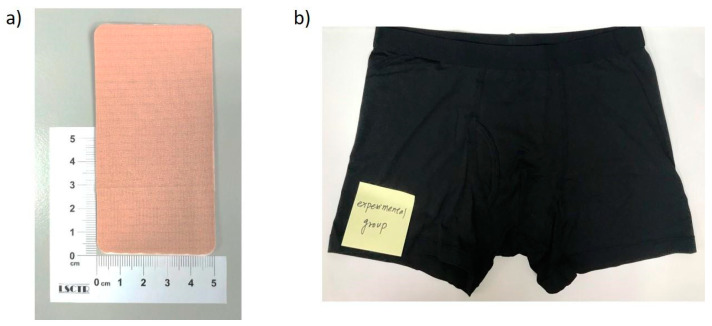
Ge-Ti-π textile with far-infrared (FIR) technology as a patch and underpants. (**a**) The Ge-Ti-π patch that was utilized for covering A-V stunt of hemodyalisis (HD) patients and (**b**) the Ge-Ti-π underpants that were used to treat vasculogenic erectile dysfunction (VED) patients.

Furthermore, using a knitting machine, the FIR fibers and general fibers were mixed and woven into a blanket that possessed an FIR radiation function; the material was called “Ge-Ti-π textile”. The FIR fibers accounted for 45–48% of the total fiber content of the 20 cm × 20 cm blanket. For the purpose of this study, the Ge-Ti-π textile was either used as a patch to cover the A-V shunt in the HD patients for up to 1 week or worn as underpants by the VED patients for up to 3 months. The Ge-Ti-π textile patch and underpants utilized to treat the HD and VED patients, respectively, are presented in Figure 1. The control groups in this study underwent treatment using either a regular textile patch, or underpants made from the same fabrics as that in the Ge-Ti-π patch or underpants, without the active FIR materials.

### 2.2. Ge-Ti-π Textile Characterization

The particle size of FIR extrudates was measured using the dynamic light scattering (DLS) method. Dawn^®^ Heleos light scatter (Wyatt Technology Corp., Santa Barbara, CA, USA) was used to perform the DLS analysis by following the protocol of NIST [48] at 25 °C. The size distribution of FIR extrudates was analyzed using Wyatt Technology’s ASTRA software (Santa Barbara, CA, USA) [49].

The emissivity of Ge-Ti-π textile was measured using a far-infrared emissivity detector TSS-5 (Japan Sensor Corporation, Tokyo, Japan). The reflection energy detection was assessed by quantifying the infrared radiation from the constant temperature emission source in which the Ge-Ti-π textile was placed. The obtained data were recorded after each calibration was completed under the heat engine for 2 h. The FIR spectral response was 2~22 µm with a measurement size and distance of 15 and 13 mm, respectively. The instrument measured emissivity in the range of 0.00~1.00.

The ash content of the FIR fiber was obtained by burning it for 3 h or longer at 575 ± 25 °C (1067 ± 5 °F). The fibers easily burned to ash at this temperature, and thus, the volatilization of inorganic compounds could be minimized. This measurement aimed to ignite all carbon compounds and then measure the inorganic minerals in the FIR fiber [50]. Thereafter, the tensile properties of the fibers were assessed according to ASTM-D2256. This method used the tensile speed of 300 ± 10 mm/min (12 ± 0.5 inch/min) with a specimen gauge length of 250 mm (10 inch) [51].

### 2.3. Color Fastness and Pilling Resistant Tests

Laundering durability of Ge-Ti-π fiber/textile was performed in SGS Taiwan Ltd. (New Taipei, Taiwan) according to the Chinese National Standards (CNS) 1494-L3027—method A-1 and CNS 8040-L3140—method A for evaluating the color fastness and pilling resistance, respectively. The CNS 1494 test is similar to the American Association of Textile Chemists and Colorists (AATCC) 61-1989 test; however, we performed the color fastness test by rinsing the fiber at 30 °C [52]. The pilling resistant test was conducted in accordance with the abovementioned standard for 5 h. The results of these assessments were evaluated based on a scale from 1 to 5, wherein 1 and 5 represented the worst and best textile quality, respectively.

### 2.4. Antibacterial and Antifungal Tests

Antibacterial and antifungal activities against *Staphylococcus aureus* (*S. aureus*; AATCC 6538) and *Trichophyton mentagrophytes* (AATCC 9533) in the Ge-Ti-π textile were evaluated by SGS Taiwan Ltd. (New Taipei, Taiwan) according to the AATCC 100:2012 and AATCC 30:2004 Part III technical manuals, respectively. To evaluate the antibacterial activity, the Ge-Ti-π textile samples were initially embedded in 1 mL of *S. aureus* bacterial suspensions with 1.0 × 10^5^–2.0 × 10^5^ CFU/mL. Six samples with a diameter of 4.8 cm were used. The samples were incubated for 24 h at 37 °C and then washed with a universal diluent. To calculate the CFU number of the bacteria at 0 and 24 h, serial dilutions of samples were placed onto agar plates and incubated for 24 h at 37 °C. After calculating the CFU number of the bacteria, the bacteria reduction rate (*R*) was determined using the equation below [53].

R(%)=100 (B−A)/B

where *A* is the total number of bacteria in the sample group recovered after 24 h of incubation, *B* is the total number of bacteria in the sample group at 0 h of incubation (control), and R is the percentage reduction in bacteria [54].

To investigate antifungal activity, 2.0 cm × 2.0 cm Ge-Ti-π textile samples were used. Sterile distilled water was prepared to inoculate 1 mL of spores with 10^5^ spores/mL concentration and then the fungal inoculum was evenly distributed onto potato dextrose agar (PDA) plates. Subsequently, a 0.05% non-ionic wetting agent (Triton X-100) was utilized to wet the Ge-Ti-π textile samples. The wet samples were placed on the PDA plates and the fungal inoculum was distributed evenly on the samples. The samples were then cultured at 27 °C for 14 days. On day 14, the degree of fungus growth over the surface of each test fabric was visually assessed to measure and record the antifungal activity [54]. The fungus growth status on the samples was categorized as “negative (no growth)”, “micro-growth”, or “massive growth”.

### 2.5. Study Design and Participants

All research protocols in this study were in accordance with the clinical trial guidelines of the code of ethics of the World Medical Association (Declaration of Helsinki). The clinical trial protocols were also approved by the Institutional Review Board (IRB No. 2-106-05-094 and 1-104-05-086) of the Tri-Service General Hospital (Taipei, Taiwan) and registered at the US National Institute of Health Clinical Trials Registry (https://clinicaltrials.gov/ct2/show/NCT04025619 and https://clinicaltrials.gov/ct2/show/NCT03359265 (accessed on 11 September 2021)). All patients undergoing maintenance HD in the HD unit and patients with VED from the outpatient department of the hospital were asked to participate in this study, and informed consent was obtained prior to the procedures.

A total of 66 HD patients aged 56–60 years, diagnosed with cardiovascular diseases including deep venous embolism of the lower extremities and valve atresia of the lower extremities, were recruited. Patients with trauma to the lower extremity for whom ligation could not be performed, who were unable express their wishes clearly or had dyslexia, and/or who were unable to walk independently were excluded from the study [55]. Further, 30 VED patients aged 40–55 years with a medical history of ED and regular sex activities were also enrolled. Patients who were heavy drinkers or smokers, unable to clearly express their wishes, or who had serious mental health issues were excluded from the study [56].

The eligible patients were randomly assigned to the experimental group (Ge-Ti-π textile treatment; *n* = 34 (HD), *n* = 21 (VED)) or the control group (regular textile treatment as placebo; *n* = 32 (HD), *n* = 9 (VED)). To specifically determine the effects of treatment in VED patients, this group was subdivided into those with mild ED symptoms (experimental group, *n* = 16; control group, *n* = 6) and severe ED symptoms (experimental group, *n* = 5; control group, *n* = 3) based on the outpatient department diagnosis database records of the patient’s medication eligibility.

### 2.6. Data Collection

#### 2.6.1. Ge-Ti-π Textile Treatment for Patients with Maintenance HD

Prior to treatment, the patients were asked to complete the visual analog scale (VAS) for pain and short-form health survey (SF-36) questionnaires. The VAS is a validated subjective measure of acute and chronic pain; it is classified into the following three categories: mild (1–3), moderate (4–6), and severe (7–10) pain [57]. The SF-36 questionnaire is often used as a measure of a person or population’s quality of life (QOL) [58]. Limb numbness/pain assessment was also performed to examine the patients’ feeling of discomfort [59]. Moreover, patients from the experimental group underwent peripheral blood flow examination including an ankle-brachial index (ABI) measurement and deep vein thrombosis evaluation (maximum venous outflow (MVO)/segmental venous capacitance (SVC)) (*n* = 20) as well as Doppler ultrasound analysis (VASOGUARDTM NICOLET Ultrasound Machine; Madison, WI, USA) (*n* = 9) after the completion of questionnaires. After 1 week of treatment, all patients were examined using the same assessments.

#### 2.6.2. Ge-Ti-π Textile Treatment for Patients with VED

Patients were provided with enough underpants (Ge-Ti-π textile underpants or regular underpants) to wear throughout the day for up to three months. Blood pressure parameters, including systolic blood pressure (SBP), diastolic blood pressure (DBP), temperature, and pulse rate were recorded prior to the treatment, and 1, 2, and 3 months following treatment to assess the safety of the applied Ge-Ti-π textile. Additionally, the penile vasculature was assessed using Doppler ultrasonography (Panther 2002 ADI, Bruel & Kjaer, Denmark) in six participants from the experimental group and three participants from the control group [56]. After giving informed consent, the participants were examined prior to the treatment, and at 1-, 2-, and 3-month follow-ups. The analyzed variable was the blood flow velocity to evaluate the effect of the treatment; the monthly changes in blood flow velocity were also compared (changes from before treatment to the first month [∆M1–0], from the first to the second month [∆M2–1], and from the second to the third month [∆M3–2]). Further, monthly evaluations of the patients’ satisfaction in quality of sexual function, and consequently, the efficacy of the treatment, were performed using the following four questionnaires associated with ED and its related effects: simplified international index of erectile function (IIEF-5), quality of erection questionnaire (QEQ), premature ejaculation diagnostic tool (PEDT), and international prostate symptom score (IPSS). The IIEF-5 is an abridged, five-item version of the 15-item IIEF that focuses on erectile function and intercourse satisfaction [60]. The IIEF-5 score, ranging from 5–25 points, classifies ED into the following five categories: none (22–25), mild (17–21), mild to moderate (12–16), moderate (8–11), and severe (5–7). The QEQ is a questionnaire that assesses satisfaction in the quality of erections that were attained and maintained and can differentiate between all ED severity groups as categorized by the IIEF-5 questionnaire scores [61]. The QEQ is evaluated as a final score that ranges from 0 to 100 points. This score is transformed on a scale of 1–30 to define the degree of ED as follows: normal (26–30), mild (22–25), mild to moderate (17–21), moderate (11–16), and severe (1–10). The PEDT is a questionnaire that standardizes and captures five main elements of the Diagnostic and Statistical Manual of Mental Disorders, Fourth Edition, Text Revision (DSM-IV-TR) in diagnosing premature ejaculation, including control, frequency, minimal sexual stimulation, distress, and interpersonal difficulty [62]. All items were scored on a five-point Likert-type scale of 0–4 points, with higher scores indicating greater sexual impairment. The IPSS is a well-known questionnaire that evaluates symptoms of prostate disease, which can cause sexual problems in men [63]. It is evaluated as a total score, which is the sum of the responses to all items, ranging from 0 (asymptomatic) to 35 (very symptomatic) points. The questionnaires’ data were generally collected through face-to-face interviews; sensitive data on sexual life were collected through self-administered questionnaires at the end of the interviews. All questionnaires’ scores were first analyzed using a trend analysis and further analyzed as mean changes after ED symptoms grouping. We defined three types of changes, namely, changes between the first and second months (∆M2–1), between the second and third months (∆M3–2), and between the first and third months (∆M3–1). Understanding the dynamic changes between each month is meaningful in determining the treatment duration that will achieve the best outcome.

### 2.7. Statistical Analysis

The data are shown as mean ± standard error of the mean. The data mean of all assessments was statistically analyzed using the Student’s “t” test for independent and paired data. The significance level was set at * *p <* 0.05, ** *p <* 0.01, and *** *p <* 0.001. Statistical calculations were performed using SPSS software version 21 (IBM SPSS, Chicago, IL, USA).

## 3. Results

### 3.1. Ge-Ti-π Textile Characterization

DLS analysis was carried out to measure the particle size of the FIR fiber. The result shows that the mean FIR fiber particle size was 49.77 ± 0.46 nm with a polydispersity index (PDI) mean of 0.21 ± 0.005. Meanwhile, the FIR emissivity detector result pointed out that the Ge-Ti-π textile has an emissivity of 0.93. The ash content percentage, strength, elongation, and coefficient of variant percentage of the Ge-Ti-π textile are presented in Table 1. In addition to several metal materials, the Ge-Ti-π textile used in this study was also made from polymers to obtain an FIR Drawn Textured Yarn (DTY) fiber. Before gaining the FIR fibers in the form of DTY fibers, the DTY firstly passed Partially Oriented Yarn (POY). In this study, the ash content of FIR fibers in the form of POY and DTY fibers was analyzed. According to the results, the FIR fibers of the Ge-Ti-π textile in the DTY form manufactured with the assistance of the fiber group had a slightly higher value (0.49) than the fibers in the POY form (0.45). This indicated that DTY fibers contained more inorganic minerals. Subsequently, the Ge-Ti-π DTY fiber was manufactured with the assistance of the process department (control) and the fiber group (experiment). Based on the results in Table 1, the Ge-Ti-π DTY fiber produced by the fiber group had greater results than the fiber produced by the process department did; the DTY fiber’s strength, elongation, and CV values were 3.05 g/d, 18.98%, and 1.21%, respectively.

### 3.2. Laundering Durability of the Ge-Ti-π Fiber and Antibacterial and Antifungal Activities of the Ge-Ti-π Textile

The results of quantitative assessments of color fastness and pilling resistance are presented in Table 2. The Ge-Ti-π fiber showed excellent color fastness compared to that shown by other fibers when rinsed at 30 °C. The Ge-Ti-π textile also possessed good pilling resistance after being washed for 5 h.

Furthermore, the Ge-Ti-π textile showed significant antibacterial properties since it obtained a bacteria reduction rate of 51.6% after being incubated with *S. aureus* for 24 h. A bacteria reduction rate between 50–90% indicates that the product has significant antibacterial efficacy [64]. Additionally, incubation with *Trichophyton mentagrophytes* for 2 weeks resulted in negative fungal growth on the Ge-Ti-π textile, indicating that the Ge-Ti-π textile could encounter and arrest fungal activity effectively.

### 3.3. Ge-Ti-π Textile Treatment for Patients with Maintenance HD

**Figure 2:**
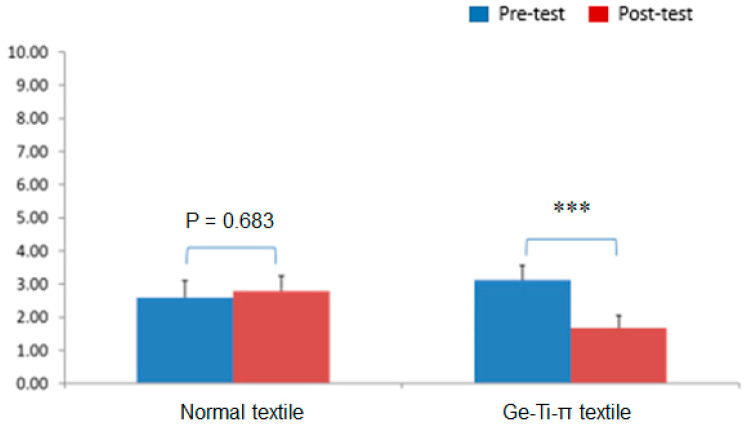
Result of the pain visual analog scale (VAS) questionnaire for patients with maintenance hemodialysis (HD) (a lower score corresponds to a reduction in pain); *** *p <* 0.001.

**Figure 3:**
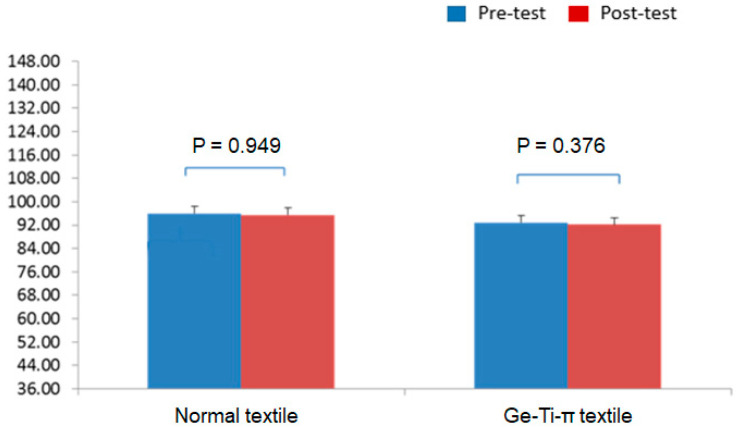
Results of the short form 36 (SF-36) health questionnaire for patients with maintenance hemodialysis (HD) (a higher score corresponds to a lower disability).

The mean age of all the HD patients was 55 years. The sex ratio was 47.1% males and 52.9% females in the experimental group, and 43.8% males and 56.3% females in the control group. All the patients completed the VAS, SF-36, and limb numbness/pain assessments. The VAS results demonstrated that the use of the Ge-Ti-π textile significantly reduced the pain score post-treatment (*p <* 0.001), while the use of the regular textile did not show any difference (Figure 2). The SF-36 revealed no statistical difference in both groups before and after treatment (Figure 3). However, the numbness/pain assessment results showed a trend similar to the VAS results, where the Ge-Ti-π treatment was observed to specifically and significantly lessen numbness and pain in the patients (Table 3). Furthermore, the peripheral blood flow examination revealed that the peripheral vascular function in the experimental group was slightly higher after treatment, albeit no significant difference was observed in particular, except for the left ankle ABI (*p <* 0.01; Table 4). Lastly, the Doppler ultrasound assessment data indicated a significant enhancement of blood flow in the right hand after 1 week of Ge-Ti-π textile treatment (*p <* 0.041; Table 5).

### 3.4. Ge-Ti-π Textile Treatment for VED Patients

The mean age and body mass index of all the VED patients were 48.6 years and 26.36 kg/m^2^, respectively. Out of the 30 recruited patients, three were unable to complete the 3-month follow-up during the observation period because of personal reasons and, hence, they were excluded. The final analysis included 19 (mild ED symptoms, *n* = 14; severe ED symptoms, *n* = 5) and 8 (mild ED symptoms, *n* = 5; severe ED symptoms, *n* = 3) patients in the experimental and control groups, respectively. Prior to the treatment, the mean SBP, DBP, temperature, and pulse rate were 130.03 mmHg, 82.40 mmHg, 36.5 °C, and 73.17 bpm, respectively. After the first month of the trial period, the mean SBP, DBP, temperature, and pulse rate were 127.10 mmHg, 79.83 mmHg, 36.37 °C, and 74.33 bpm, respectively. After the second month of the trial period, the mean SBP, DBP, temperature, and pulse rate were 126.33 mmHg, 80.37 mmHg, 36.46 °C, and 74.40 bpm, respectively. Finally, after the third month of trial period, the mean SBP, DBP, temperature, and pulse rate were 127.26 mmHg, 81.33 mmHg, 36.39 °C, and 74.48 bpm, respectively. Overall, these results demonstrated that the body temperature was within the normal range for all the participants and the blood pressure parameters were similar between the two groups, thereby indicating that the Ge-Ti-π underpants treatment was safe with no adverse effects being observed.

**Figure 4:**
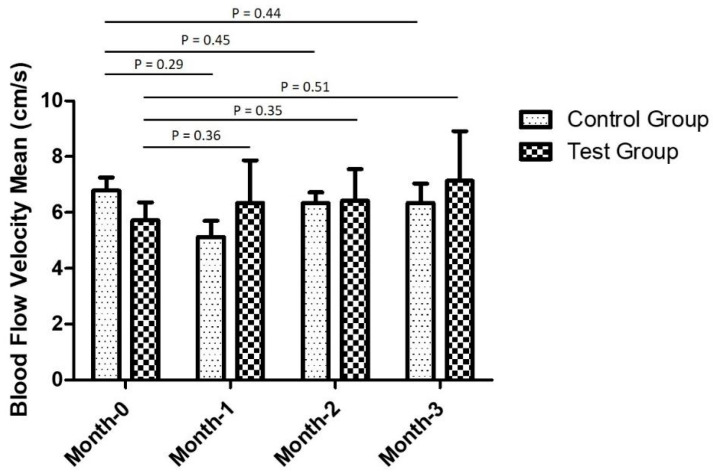
Doppler ultrasonography results of vasculogenic erectile dysfunction (VED) patients in terms of monthly penile blood flow velocity means (cm/s) in the control and the experimental groups.

Subsequently, Doppler ultrasonography demonstrated comparable differences in penile blood flow velocity between both groups as time progressed. The blood flow velocity was represented by the measurement of the S (peak systolic velocity) parameter in the ultrasonography images. In the control group, the average blood flow velocity of three VED patients prior to treatment and at the 1-, 2-, and 3-month follow-ups were 6.79, 5.13, 6.34, and 6.35 cm/s, respectively, whereas in the experimental group, the average blood flow velocity of six patients were 5.72, 6.35, 6.42, and 7.14 cm/s, respectively (Figure 4). Although there was no statistically significant finding on a month-to-month basis, the experimental group demonstrated a rising trend in the blood flow velocity from before the treatment to the third month of the trial. In contrast, the penile blood flow velocity in the VED patients treated with regular underpants dropped after 1 month of the study and climbed again after the second month of the trial.

Additionally, the monthly alterations in the mean penile blood flow velocity in the VED patients are represented in Table 6. The changes in the average penile blood flow velocity in the experimental group tended to increase every month, although the improvement differences were not statistically significant, as shown in the *p*-values. Nevertheless, they indicated positive changes in the VED patients 3 months after treatment using the Ge-Ti-π underpants. In contrast, in the control group, the changes in the average penile blood velocity fluctuated every month, and there was a substantial dip at the 1-month follow-up among those treated with regular underpants.

**Figure 5:**
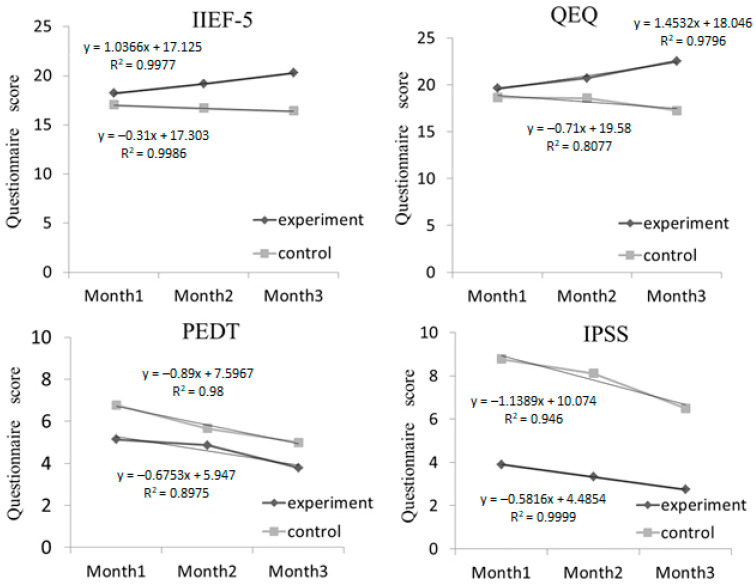
Trend analysis of all questionnaires for patients with mild vasculogenic erectile dysfunction (VED) symptoms.

**Figure 6:**
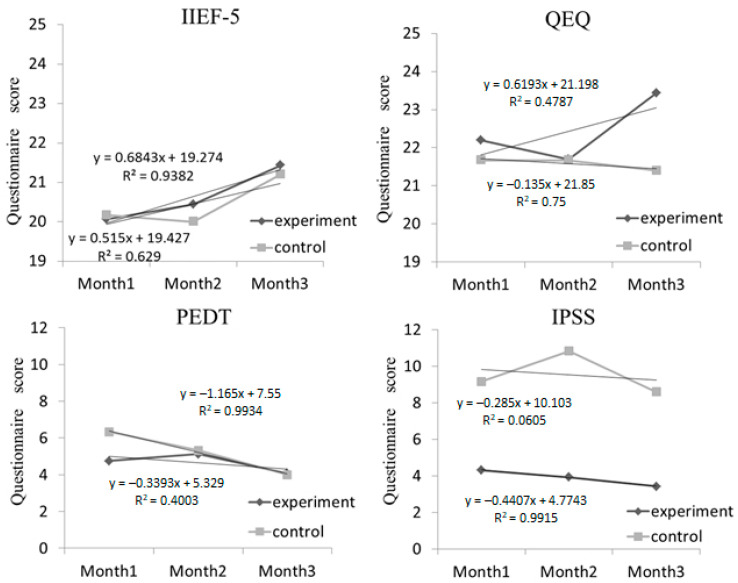
Trend analysis of all questionnaires for patients with severe vasculogenic erectile dysfunction (VED) symptoms.

Furthermore, the scores obtained from all of the questionnaires were investigated by using the trend analysis; in this case, the IIEF-5 and QEQ questionnaires were related to satisfaction in the quality of sexual function, while the PEDT and IPSS questionnaires were related to the ED indications. The results indicated significant positive trends were remarkably indicated in the experimental group for mild and severe ED symptoms; the scores in the experimental group were higher than those in the control group for the IIEF-5 and QEQ questionnaires and were lower than those in the control group for the PEDT and IPSS questionnaires, respectively (Figure 5 and Figure 6).

The change in the mean of the questionnaire scores for patients with mild and severe ED symptoms is presented in Table 7. The mean scores of all the questionnaires for each month are useful in determining the appropriate duration of treatment that achieves the best outcome. As shown in Table 7, the patients with mild ED symptoms had no significant difference in their scores regardless of the treatment group. However, the patients with severe ED symptoms in the experimental group had significantly different QEQ questionnaire scores (*p <* 0.05) at (ΔM3–1), compared with those in the control group. This result indicated that the use of the Ge-Ti textile remarkably enhanced the quality of sexual function in the patients with severe ED symptoms after a 3-month trial period. The experiment group showed better results than the control group in the IIEF-5, PEDT, and IPSS questionnaire scores, although the differences were not statistically significant.

## 4. Discussion

FIR constitutes the electromagnetic spectrum between a wavelength of 15 μm and 1 mm [9]. Its biological effects and biomedical applications have been widely investigated [10]. The Ge-Ti-π textile developed as a polymer composite and investigated in this study is believed to be able to emit FIR. The study results show that the Ge-Ti-π textile possesses high FIR emissivity (0.93), indicating that the material can effectively radiate its contained energy in the FIR spectrum [65]. Meanwhile, the Ge-Ti-π fibers were observed to have nano-sized particles; the nano size of the particles may greatly impact the emission property of the Ge-Ti-π textile. Based on a previous study, a decrease in the particle size in tourmaline superfine powder increased the powder’s FIR emissivity [66].

The FIR fibers of Ge-Ti-π textile used in this study was produced in the form of DTY fibers because yarn has a slightly higher ash content in the DTY form than in the POY form. This indicated that the Ge-Ti-π textile in the DTY form had a slightly higher inorganic mineral density [67]. A previous study also showed that a high ash content was proportional to the increment of thermal stability [68]. Additionally, according to the results of the tenacity measurement, the DTY fibers manufactured with the assistance of the fiber group are stronger and more ductile with more uniform material contents [69] than those generated with the assistance of the process department.

Ceramic particles, such as TiO_2_, are known to imbue textiles with antibacterial properties [40,41]. The Ge-Ti-π textile, which contained titanium as one of the fillers, exhibited a significant efficacy against *S. aureus* bacteria [64]. The incorporation of TiO_2_ in polyester is shown to provide antibacterial and self-cleaning properties to the textile [39,40]. The Ge-Ti-π textile also showed negative mold growth after 14 days of fungal incubation. This result is in accordance with a study by Behzadnia et al., which showed that the addition of ceramic TiO_2_ to wool resulted in the fabric possessing antifungal properties [70].

FIR radiation has many advantages for health. In this study, the benefits of the Ge-Ti-π textile in treating HD and VED patients were investigated. The use of the Ge-Ti-π textile as a treatment for patients with maintenance HD and VED demonstrated positive results. In addition to reducing limb numbness and pain and significantly enhancing blood circulation, the Ge-Ti-π textile was shown to be safe and produced no adverse effects in long-term use.

The notable findings in this study on HD patients included a significant reduction in the post-treatment pain score (*p <* 0.001) and limb numbness/pain score (*p <* 0.001) assessed using the VAS and limb numbness/pain questionnaires, respectively, and a notable increase in the blood flow of the right hand in ~12.23% of patients after being treated using the Ge-Ti-π patch for a week. These results correspond to those of a previous study conducted by Szu-Chia et al., who observed an association between FIR and ABI in HD patients [71].

Furthermore, this study revealed that the quality of sexual function was significantly improved in the VED patients, particularly in the patients with severe symptoms, based on the scores of the IIEF-5 and QEQ questionnaires. Although the PEDT and IPSS questionnaires revealed no statistically significant difference in the premature ejaculation and prostate problem investigation, the scores in the experiment group were observed to be consistently lower than those in the control group. Further, the notable difference in the questionnaire scores between patients with mild and severe ED symptoms with an increase in the trial duration demonstrated that the indications were perceived less by the patients who underwent treatment using the Ge-Ti-π underpants. The experimental group of VED patients also showed positive trends for an improvement in penile blood circulation after using the Ge-Ti-π underpants for 3 months. Based on the Doppler ultrasonography data, the experimental group experienced an almost 25% increase in the blood flow velocity after treatment completion, even though the improvement was not statistically significant. These results were consistent with previous findings, wherein FIR material-incorporated textiles effectively improved blood circulation and metabolism [8,14,25,72,73].

This study was the first of its kind to apply sports products across domains for the treatment of vascular dysfunctions without using medication intervention, indicating the novelty of the study. Additionally, the protocol of the clinical trial was designed to be prescient, and the intervention in the individual participants in this study was observed to have sufficient efficacy.

We acknowledge the study’s limitations regarding VED-related sexual function assessment. Since the vast majority of men in this study did not report ejaculatory volume, our findings predominantly reflected the perspectives of men with normal ejaculatory function. If men are symptomatic, their sexual partners might have different attitudes toward their ejaculatory function. Other considerations include regional and volunteer bias. While we did not collect the participants’ specific geographic information, we can assume that most participants came from Taiwan’s northwestern region. Due to geographic differences in sexual behaviors and attitudes, these findings may be limited in their applicability. Additionally, functional FIR textile technology is currently limited by the lack of reputable support for the proposed therapeutic effects of FIR in scientific research and clinical trials. For this reason, the use of FIR textiles is still considered as an alternative therapy rather than as a form of mainstream health and medical treatment. Hence, more comprehensive and tailored trials are required to support viable clinical results.

The benefits of FIR textiles for non-therapeutic applications are significant and relatively well-demonstrated. However, the quantity of FIR-active material that is incorporated into the fiber, which determines the level of FIR that is directed back toward the body, is a limitation of the current FIR textile products and technologies. Not only can ceramic pores be blocked by dirt and sweat, but repeated washing can also damage the ceramic layers and reduce its functionality. Hence, manufacturers recommend current FIR textiles to be washed at temperatures below 30 °C and without the use of fabric softeners. This study showed that the novel Ge-Ti-π fiber has good pilling resistance and excellent color fastness when washed at 30 °C. The presented innovative textile technology not only restricts the reduction in the quantity of Ge-Ti metals used but also facilitates the extension of the service life of the functional FIR textile and allows it to tolerate high-temperature washes. It is anticipated that this technology will facilitate the production of high-quality and inexpensive functional textiles that patients will be more willing to wear. Concisely, the trial protocol and FIR technology introduced in our study provide a non-invasive and complementary treatment option for maintaining physiological functions.

## 5. Conclusions

In conclusion, we developed a novel FIR fiber named Ge-Ti-π textile from germanium, titanium, and several polymers. This strong and ductile fabric showed effective FIR emissivity, remarkable color fastness and pilling resistance, and outstanding antibacterial and antifungal properties. In addition, we demonstrated that the use of the Ge-Ti-π textile can produce potential and beneficial effects for patients with maintenance HD and VED by reducing physical discomfort and enhancing blood circulation. Moreover, Ge-Ti-π textile treatment may improve the quality of sexual function in VED patients. It is a safe and affordable treatment option. While further clarification is required in future studies, our results suggest that the integration of a routine medical prescription with complementary alternative treatments has the potential to significantly improve the clinical outcomes of these patients without cardiovascular risks.

## 6. Patents

The work reported in this manuscript has resulted in several patents in Taiwan and Japan, they are “Far-Infrared Fiber and Its Composition and Application” [Taiwan Patent No. I673406 and Japan Patent No. 6718490], “Far-Infrared Underpants” [Taiwan Patent No. M560198], and “Underpants of Far-Infrared Fiber for Enhancing the Male Sexual Function” [Japan Patent No. 6464245].

## Figures and Tables

**Table 1:** The results of ash content percentage, strength, elongation, and coefficient of variant assessments of Ge-Ti-π textile.

		Ge-Ti-π Textile (in POY; Control)	Ge-Ti-π Textile (in DTY; Experimental)
Ash Content (%)		0.45	0.49
	DTY Fiber Strength (g/d)	Elongation (%)	CV (%)
Ge-Ti-π textile (with DTY manufactured by process department; control)	2.19	15.31	4.00
Ge-Ti-π textile (with DTY manufactured by fiber group; experimental)	3.05	18.98	1.21

POY: Partially Oriented Yarn; DTY: Drawn Textured Yarn; CV: Coefficient of Variant.

**Table 2:** The quantitative results of color fastness and pilling resistance evaluation of the Ge-Ti-π fiber.

Sample (Product)	Degree
Color Fastness Test	
Ge-Ti-π fibers	4–5
Acetate fibers	4–5
Cotton fibers	4–5
Nylon fibers	4–5
Polyester fibers	4–5
Acrylic fibers	4–5
Wool fibers	4–5
Pilling Resistance Test	
Ge-Ti-π fibers	4

**Table 3:** Prevalence of limb numbness and discomfort in patients with maintenance HD before and after Ge-Ti-π textile treatment.

	Normal Textile			Ge-Ti-π Textile		
	Pre-Test	Post-Test	*p*-Value	Pre-Test	Post-Test	*p*-Value
*n*	32			34		
Numbness+	10 (31)	9 (28)	***	11 (32)	6 (18)	***
Discomfort+	13 (41)	12 (38)	***	14 (41)	9 (26)	***

Values in parentheses represent percentages; *** *p <* 0.001.

**Table 4:** Comparison of peripheral vascular function for patients with maintenance HD before and after Ge-Ti-π textile treatment.

	Pre-Test	Post-Test	*p*-Value
	Mean ± SE	Mean ± SE	
R Brachial ABI	0.99 ± 0.01	0.99 ± 0.01	0.584
L Brachial ABI	0.97 ± 0.01	0.97 ± 0.01	0.701
R Thigh ABI	1.09 ± 0.06	1.13 ± 0.04	0.243
L Thigh ABI	1.16 ± 0.03	1.19 ± 0.03	0.352
R BI Knee ABI	1.09 ± 0.06	1.12 ± 0.03	0.216
L BI Knee ABI	1.14 ± 0.05	1.15 ± 0.04	0.369
R Ankle ABI	1.08 ± 0.04	1.10 ± 0.04	0.507
L Ankle ABI	1.08 ± 0.04	1.11 ± 0.04	0.010 **
Avg R MVO/SVC	0.74 ± 0.03	0.78 ± 0.04	0.206
Avg L MVO/SVC	0.73 ± 0.04	0.74 ± 0.04	0.785

R: right; L: left; ABI, Ankle-Brachial Index; Avg, average; MVO, maximum venous outflow; SVC, segmental venous capacitance; ** *p <* 0.01.

**Table 5:** Comparison of Doppler ultrasound analysis for patients with maintenance HD before and after Ge-Ti-π textile treatment.

Blood Flow	Pre-Test	Post-Test	*p*-Value
	Mean ± SE (cm/s)	Mean ± SE (cm/s)	
Right Hand	25.76 ± 2.02	28.91 ± 2.17	0.041 *
Left Hand	20.65 ± 1.88	26.42 ± 1.84	0.056

* *p <* 0.05.

**Table 6:** Compendium of the monthly change of the average penile blood flow velocity in the VED patients.

ΔMonth	Experiment				Control			
	*n*	Mean Age (Years)	Blood Flow Velocity Change (Mean ± SE (cm/s))	*p*-Value	*n*	Mean Age (Years)	Blood Flow Velocity Change (Mean ± SE (cm/s))	*p*-Value
ΔM1-0	6	53.5 ± 0.19	0.64 ± 0.72	0.36	3	49.67 ± 0.09	−1.67 ± 1.33	0.29
ΔM2-1			0.06 ± 0.69	0.48			1.22 ± 0.88	0.36
ΔM3-2			0.72 ± 1.27	0.25			0.01 ± 0.61	0.5

∆M1-0, changes from before treatment to the first month; ∆M2-1, changes from the first to second month; ∆M3-2, changes from the second to third month.

**Table 7:** Summary of changes in the mean scores of all questionnaires in the VED patients.

	ΔMonth	*n*	IIEF-5			QEQ			PEDT			IPSS		
			Control	Experiment	*p*	Control	Experiment	*p*	Control	Experiment	*p*	Control	Experiment	*p*
Mild Symptoms	ΔM2-1	22	−0.67 ± 2.17	1.50 ± 1.50	0.447	0.00 ± 2.72	−1.67 ± 2.51	0.712	−5.00 ± 4.47	1.88 ± 1.88	0.106	4.76 ± 7.69	−1.07 ± 1.35	0.260
	ΔM3-2	19	0.80 ± 1.50	2.86 ± 2.50	0.641	−2.00 ± 3.09	4.29 ± 4.47	0.432	−4.00 ± 5.34	−4.29 ± 2.22	0.954	−3.43 ± 1.67	−2.65 ± 2.15	0.840
	ΔM3-1	19	0.00 ± 1.26	4.00 ± 1.73	0.205	0.00 ± 1.05	1.67 ± 3.40	0.778	−5.00 ± 5.70	−2.50 ± 2.15	0.615	−6.29 ± 2.91	−3.88 ± 2.59	0.616
Severe Symptoms	ΔM2-1	8	−2.67 ± 14.11	11.20 ± 4.63	0.292	−1.11 ± 1.11	20.67 ± 10.61	0.175	−6.67 ± 19.22	−12.00 ± 11.47	0.806	−15.24 ± 16.69	−3.43 ± 4.98	0.427
	ΔM3-2	8	−6.67 ± 5.33	8.00 ± 4.20	0.075	−6.67 ± 10.18	8.00 ± 3.74	0.153	1.67 ± 7.27	−5.00 ± 9.49	0.644	−0.95 ± 0.92	−1.71 ± 1.14	0.162
	ΔM3-1	8	−9.33 ± 19.23	19.20 ± 5.85	0.126	−7.78 ± 9.88	28.67 ± 9.35	0.045 *	−5.00 ± 13.23	−17.00 ± 7.17	0.411	−14.29 ± 17.22	−5.14 ± 4.46	0.535

∆M2-1, changes from the first to second month; ∆M3-2, changes from the second to third month; ∆M3-1, changes from the first to third month; IIEF-5, simplified international index of erectile function; QEQ, quality of erection questionnaire; PEDT, premature ejaculation diagnostic tool; IPSS, international prostate symptom score; * *p <* 0.05.

## Data Availability

The data used to support the findings of this study are available from the corresponding author upon request.
